# The role of anti-citrullinated protein antibody reactivities in an inception cohort of patients with rheumatoid arthritis receiving treat-to-target therapy

**DOI:** 10.1186/s13075-018-1635-7

**Published:** 2018-07-13

**Authors:** Maria Karolina Jonsson, Aase Haj Hensvold, Monika Hansson, Anna-Birgitte Aga, Joseph Sexton, Linda Mathsson-Alm, Martin Cornillet, Guy Serre, Siri Lillegraven, Bjørg-Tilde Svanes Fevang, Anca Irinel Catrina, Espen Andre Haavardsholm

**Affiliations:** 1 Department of Rheumatology, Haukeland University Hospital, Pb 1400, NO-5021 Bergen, Norway; 2 Department of Clinical Science, University of Bergen, Bergen, Norway; 3 Center for Molecular Medicine, Karolinska University Hospital, Stockholm, Sweden; 4 Department of Rheumatology, Diakonhjemmet Hospital, Oslo, Norway; 5 Thermo-Fisher Scientific, Uppsala, Sweden; 6 Epithelial Differentation and Rheumatoid Autoimmunity Unit, UMRS 1056 Inserm University of Toulouse, Toulouse, France; 7 Department of Health and Society, Oslo University Hospital, Oslo, Norway

**Keywords:** Rheumatoid arthritis, Biomarkers, Inflammation, Imaging, Outcomes

## Abstract

**Background:**

Anti-citrullinated protein antibody (ACPA) reactivities precede clinical onset of rheumatoid arthritis (RA), and it has been suggested that ACPA reactivities towards distinct target proteins may be associated with differences in RA phenotypes. We aimed to assess the prevalence of baseline ACPA reactivities in an inception cohort of patients with early RA, and to investigate their associations with disease activity, treatment response, ultrasound findings and radiographic damage.

**Methods:**

Disease-modifying antirheumatic drug (DMARD)-naïve patients with early RA, classified according to the 2010 American College of Rheumatology (ACR)/European League Against Rheumatism (EULAR) criteria, were included in the ARCTIC trial and assessed in the present analysis. During follow up, patients were monitored frequently and treatment was adjusted according to a predetermined protocol, starting with methotrexate monotherapy with prednisolone bridging. Analysis of 16 different ACPA reactivities targeting citrullinated peptides from fibrinogen, alpha-1 enolase, vimentin, filaggrin and histone was performed using a multiplex chip-based assay. Samples from 0, 3, 12 and 24 months were analysed. Controls were blood donors with similar characteristics to the patients (age, gender, smoking status).

**Results:**

A total of 217 patients and 94 controls were included. Median [25, 75 percentile] number of ACPA reactivities in all patients was 9 [4, 12], and were most prevalent in anti-cyclic citrullinated peptide /rheumatoid factor-positive patients 10 [7, 12]. Disease activity measures and ultrasound scores at baseline were lower in ACPA reactivity-positive compared to ACPA reactivity-negative patients. ACPA reactivity levels decreased after 3 months of DMARD treatment, most pronounced for fibrinogenβ 60–74 to 62% of baseline antibody level, with least change in filaggrin 307–324 to 81% of baseline antibody level, both *p* < 0.001. However, outcomes in disease activity measures, ultrasound and radiographic scores after 12 and 24 months were not associated with baseline levels or changes in ACPA reactivity levels and/or seroreversion after 3 months.

**Conclusions:**

The clinical relevance of analysing ACPA reactivities in intensively treated and closely monitored early RA was limited, with no apparent associations with disease activity, prediction of treatment response or radiographic progression. Further studies in larger patient materials are needed to understand the role of ACPA reactivities in patients with RA classified according to the 2010 ACR/EULAR criteria and treated according to modern treatment strategies.

**Trial registration:**

www.ClinicalTrials.gov, NCT01205854. Registered on 21 September 2010.

**Electronic supplementary material:**

The online version of this article (10.1186/s13075-018-1635-7) contains supplementary material, which is available to authorised users.

## Background

A high level of anti-citrullinated protein antibodies (ACPA) is predictive of radiographic progression in rheumatoid arthritis (RA) [1, 2], and ACPA positivity has been associated with radiographic damage even before RA onset [1] and in early RA [1, 3–7]. Positivity to the anti-cyclic citrullinated peptide (anti-CCP2) test, hereafter referred to as “anti-CCP”, reflects presence of antibodies to mixed cyclic citrullinated peptides (i.e. ACPAs) as an artificial mimic of the true autoantigens [8]. The ACPA response against citrullinated antigens in RA, hereafter referred to as “ACPA reactivity”, has been shown to be heterogeneous [9–15]. Presence of ACPA reactivities may precede the onset of anti-CCP positivity [16], as several studies have shown that both the number of ACPA reactivities and their individual titres increase before clinical onset of RA [12, 13, 16–19].

RA patients may be characterised by distinct autoantibody profiles, as serum samples from the majority of anti-CCP-positive patients react with one or more specific citrullinated target proteins [12, 13, 16, 17, 20]. ACPA reactivities have also been found in patients who are anti-CCP negative [14, 15, 20–22]. Previous studies have shown that the individual titre of selected ACPA reactivities, such as anti-citrullinated vimentin, declines significantly after initiation of disease-modifying antirheumatic drug (DMARD) treatment, while the presence of anti-CCP antibodies remains stable over time [23, 24]. Higher numbers of specificities have been associated with increased risk of relapse in patients with early RA, who are in clinical remission and tapering DMARDs [25].

ACPA reactivity against citrullinated vimentin has been proposed to be involved in the bone-destructive processes in undifferentiated arthritis [26], early RA [23, 24, 26, 27] and established RA [28]. In early RA, seroreversion of citrullinated vimentin during the first 3 months of treatment has been shown to be associated with significantly less 2-year radiographic progression, compared with patients who remained positive [23]. However, the clinical relevance of measuring ACPA reactivities to obtain prognostic information on treatment response or radiographic damage in early undifferentiated arthritis [22, 29–31] or early RA [32] has not been established.

To our knowledge, the associations between individual ACPA reactivities and disease characteristics have not been studied in patients with early RA classified according to the 2010 American College of Rheumatology (ACR)/European League Against Rheumatism (EULAR) criteria. The aim of this study was to assess the prevalence of selected baseline ACPA reactivities, and to investigate the association between ACPA reactivities and disease activity, ultrasound findings, treatment response and radiographic damage in an inception cohort of patients with early RA.

## Methods

### Design and setting of the study

We used data from the completed “Aiming for remission in rheumatoid arthritis: a randomized trial examining the benefit of ultrasound in a clinical tight control regimen” (the ARCTIC trial, NCT01205854) [33], a study designed to assess whether incorporation of ultrasound information into treatment decisions would lead to improved patient outcomes. All patients were treated according to a tight-control treat-to-target strategy, with evaluation at baseline and 12 additional study visits during the 2-year follow up. The treatment target was no swollen joints and Disease Activity Score (DAS) < 1.6 [34], and in half of the patients an additional target was no joints with disease activity demonstrated on power Doppler ultrasound [33]. Core disease activity measures were collected at each visit. Initial treatment consisted of methotrexate monotherapy 15 mg/week escalating to 20 mg/week and prednisolone starting at 15 mg with tapering to stop over 7 weeks. If the treatment target was not achieved, treatment was intensified following a predetermined treatment protocol, with escalation to triple therapy and then biologic DMARDs (bDMARDs). Swollen joints and/or joints with power Doppler ultrasound activity could be injected with triamcinolone hexacetonide (up to a maximum of 80 mg per visit). As clinical and radiographic outcomes of the two strategy arms were similar after 2 years, in the current report the data from the two arms were pooled and analysed together.

### Patients and controls

In the ARCTIC trial [33], 230 DMARD-naïve patients with early RA classified according to the 2010 ACR/EULAR criteria were recruited at 11 Norwegian rheumatology centres between September 2010 and April 2013. The patients were 18–75 years of age with symptom duration less than 2 years from first patient-reported swollen joint, and DMARD-naïve with indication for DMARD treatment. For the current analyses, all patients with follow-up biobank serum samples were selected (*n* = 217). Controls were blood donors with similar characteristics to the patients included in the study (age, gender and smoking status (*n* = 94)). Serum samples were collected at each visit and stored at - 70 °C. The study was approved by the local ethics committee of the South-Eastern Norway Regional Health Authority and was conducted in compliance with the Declaration of Helsinki and the International Conference on Harmonization Guidelines for Good Clinical Practice. All patients provided written informed consent.

### Laboratory examinations

Analysis of 16 ACPA reactivities and the corresponding native arginine-containing control peptides targeting citrullinated peptides from fibrinogen (Fib), alpha-1 enolase (citrullinated enolase peptide 1 (CEP-1)), vimentin (Vim), filaggrin (Fil) and histone (H) was performed using a multiplex chip-based assay based on the ImmunoCAP ISAC system (Phadia AB, Uppsala, Sweden) [15, 20]. All samples from 0, 3, 12 and 24 months were analysed. ACPA reactivity titres (AU/ml) were considered positive if above the 98-percentile of values in 619 subjects without RA [20]. Erythrocyte sedimentation rate (ESR) and C-reactive protein (CRP) were analysed locally by in-house standard methodology. Anti-CCP was analysed by fluorometric enzyme immunocapture assay (FEIA) (positive if ≥ 10 IU/mL) and rheumatoid factor (RF) by ELISA (positive if ≥ 25 IU/mL).

### Clinical and imaging assessments

Clinical joint examination was performed using the 44 swollen joint count (SJC44) and Ritchie Articular Index for tender joints [35]. Patients and physicians reported the overall assessment of disease activity and pain on visual analogue scales (VAS), range 0–100. The composite index DAS was calculated [34] and response according to the EULAR criteria [36] and fulfillment of ACR/EULAR Boolean remission criteria were evaluated [37].

Radiographic examinations of the hands and feet from baseline, 12 and 24 months were scored according to the van der Heijde modified Sharp score [38]. Scoring was performed in chronological order by two trained readers blinded to clinical information, and the average of the two scores was used. Presence of erosive disease was defined as van der Heijde modified Sharp erosion score ≥ 3, in line with the definition suggested by a EULAR task force [39]. Radiographic progression was defined as a change in van der Heijde modified Sharp total score of ≥ 2 units over 2 years, which is above the smallest detectable change (1.94 units).

Ultrasound examination was performed according to a validated semi-quantitative 32-joint protocol with scores of 0–3, separately for grey scale synovitis and power Doppler ultrasound [40]. Half the patients underwent ultrasound examination at all visits, while the patients in the conventional group were examined clinically at every visit and by ultrasound at baseline, 12 and 24 months [33]. Examiners were thoroughly trained and an atlas was available for reference [40].

### Statistical analysis

Baseline characteristics were compared using the chi-square test, *t* test and Mann-Whitney U test, as appropriate. Correlation between anti-CCP/ACPA reactivity levels and number of ACPA reactivities and disease activity measures, ultrasound and radiography scores were assessed using Spearman’s rank correlation coefficient. Spearman’s correlation was classified as very weak, weak, moderate, strong or very strong [41]. Association between number of ACPA reactivities and treatment response was evaluated using the Mann-Whitney U test. Association between anti-CCP/ACPA reactivity status and continuous variables was evaluated by the Mann-Whitney U test, and association with categorical variables was assessed by the chi-square test. ACPA reactivity median levels at baseline and follow-up visits were compared by paired samples using the Wilcox test, comparing each time point with the baseline level. A *p* value <0.05 was considered statistically significant. Statistical analyses were performed using Stata Statistical Software, version 14 (StataCorp LLC, TX, USA) and R Statistical Software, version 3.4.0 (copyright 2017, The R Foundation for Statistical Computing).

## Results

### Patient characteristics

**Fig. 1:**
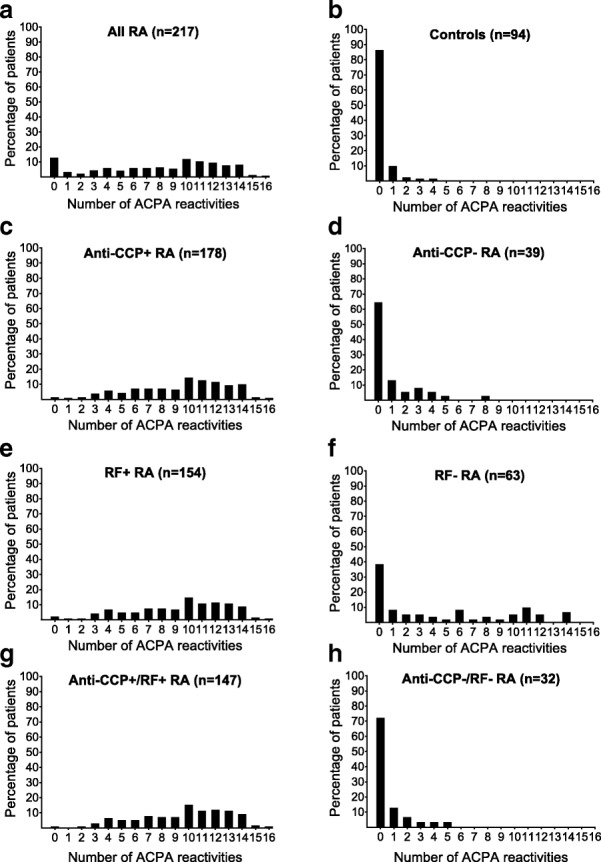
Number of anti-citrullinated protein antibody (ACPA) reactivities according to autoantibody status. **a** All patients with rheumatoid arthritis (RA). **b** Controls. **c** Patients with anti-cyclic citrullinated peptide (anti-CCP)+ RA. **d** Patients with anti-CCP- RA. **e** Patients with rheumatoid factor (RF)+ RA. **f** Patients with RF- RA. **g** Patients with anti-CCP+/RF+ RA. **h** Patients with anti-CCP-/RF- RA

A total of 217 patients and 94 healthy controls were included in the study. Baseline characteristics according to autoantibody subgroups are provided in Table 1. Presence of ACPA reactivities was seen mainly in patients positive for anti-CCP and RF (Table 1), but ACPA reactivities also occurred more frequently in anti-CCP/RF negative patients than in controls (0 (0, 1) vs. 0 (0, 0), *p* = 0.050; Table 1, Fig. 1). The anti-CCP level significantly correlated with the number of citrullinated antigens recognised (*r* = 0.76, *p* < 0.0001). ACPA reactivity presence in the RF-negative subset was generally higher than in the anti-CCP-negative group (Table 1). Among the 63 RF-negative patients, 31 were anti-CCP positive.

**Table 1:** Baseline characteristics and anti-citrullinated protein antibody (ACPA) reactivities in subgroups of patients with rheumatoid arthritis (RA) and controls

	All RA *n* = 217	Anti-CCP+ *n* = 178	Anti-CCP- *n* = 39	RF+ *n* = 154	RF- *n* = 63	Anti-CCP+/RF+ *n* = 147	Anti-CCP-/RF- *n* = 32	Controls *n* = 94
Age, years^a^	51.5 (13.6)	50.8 (13.2)	55.0 (14.9)	51.9 (13.3)	50.8 (14.2)	51.7 (13.6)	55.0 (16.1)	52.1 (9.2)
Female^b^	131 (60)	109 (61)	22 (56)	91 (59)	40 (63)	86 (59)	17 (53)	57 (61)
Ever-smoker^b^	148 (68)	122 (69)	26 (67)	109 (71)	39 (62)	103 (70)	20 (62)	60 (64)
DAS^a^	3.5 (1.2)	3.4 (1.1)	4.0 (1.3)	3.5 (1.2)	3.5 (1.2)	3.42 (1.1)	3.9 (1.2)	NA
DAS28^a^	4.4 (1.2)	4.4 (1.2)	4.7 (1.2)	4.5 (1.2)	4.2 (1.3)	4.5 (1.1)	4.5 (1.1)	NA
vdHSS total^c^	4.0 [1.5, 8.0]	4.0 [1.5, 7.9]	4.5 [2.0, 10]	4.5 [2.0, 8.0]	3.5 [1.5, 10]	4.5 [2.0, 8.0]	5.5 [1.8, 12.8]	NA
vdHSS erosion^c^	3.0 [1, 4.5]	3.0 [1, 4.5]	3.0 [1.0, 5.5]	3.0 [1.5, 4.5]	3.0 [1.0, 5.5]	3.0 [1.0, 4.5]	3.0 [1.0, 6.3]	NA
vdHSS JSN^c^	1.0 [0.0, 3.0]	1.0 [0.0, 3.0]	1.5 [0.0, 5.0]	1.0 [0.0, 3.0]	1.0 [0.0, 3.0]	1.0 [0.0, 3.0]	1.5 [0.0, 6.5]	NA
Ultrasound grey scale^c^	18 [10, 28]	16 [9, 24]	33 [20, 51]	17 [10, 26]	21 [12, 36]	16 [9, 25]	33 [21, 52]	NA
Ultrasound power Doppler^c^	7 [3, 14]	6 [2, 12]	14 [6, 28]	6 [2, 13]	8 [3, 15]	6 [2, 12]	13 [6, 29]	NA
Number of ACPA reactivities^c^	9 [4, 12]	10 [7, 12]	0 [0, 1]	10 [7, 12]	2 [0, 10]	10 [7, 12]	0 [0, 1]	0 [0, 0]
ACPA reactivity status, *n* (%)								
Fibβ 60-74cit	162 (75)	160 (90)	2 (5)	136 (88)	26 (41)	134 (91)	0 (0)	0 (0)
Vim 60-75cit	159 (73)	152 (85)	7 (18)	130 (84)	29 (46)	128 (87)	5 (16)	5 (5)
H4 31-50cit	145 (67)	142 (80)	3 (8)	119 (77)	26 (41)	118 (80)	2 (6)	1 (1)
CEP-1	140 (65)	137 (77)	3 (8)	117 (76)	23 (37)	115 (78)	1 (3)	1 (1)
Fil 307-324cit	136 (63)	134 (75)	2 (5)	113 (73)	23 (37)	112 (76)	1 (3)	0 (0)
Fibα 573cit	123 (57)	121 (68)	2 (5)	99 (64)	24 (38)	99 (67)	2 (6)	0 (0)
Fibβ 36-52cit	117 (54)	116 (65)	1 (3)	96 (62)	21 (33)	96 (65)	1 (3)	2 (2)
H3 1-30cit	107 (49)	106 (60)	1 (3)	93 (60)	14 (22)	92 (63)	0 (0)	0 (0)
H4 14-34cit	105 (48)	103 (58)	2 (5)	90 (58)	15 (24)	88 (60)	0 (0)	3 (3)
H3 21-44cit	96 (44)	94 (53)	2 (5)	80 (52)	16 (25)	79 (54)	1 (3)	1 (1)
Fibα 621-635cit	93 (43)	92 (52)	1 (3)	78 (51)	15 (24)	77 (52)	0 (0)	1 (1)
Vim 2-17cit	88 (41)	87 (49)	1 (3)	80 (52)	8 (13)	79 (54)	0 (0)	0 (0)
Fibα 36-50cit	79 (36)	79 (44)	0 (0)	67 (44)	12 (19)	67 (46)	0 (0)	0 (0)
Fibα 591cit	69 (32)	66 (37)	3 (8)	56 (36)	13 (21)	55 (37)	2 (6)	1 (1)
Fibβ 74cit	66 (30)	60 (34)	6 (15)	54 (35)	12 (19)	51 (35)	3 (9)	3 (3)
Fibβ 72cit	27 (12)	24 (13)	3 (8)	21 (14)	6 (1)	20 (14)	2 (6)	2 (2)

*Abbreviations*: *RA* rheumatoid arthritis; *anti-CCP* anti-cyclic citrullinated peptide, *RF* rheumatoid factor, *DAS* Disease Activity Score, *vdHSS* van der Heijde modified Sharp score, *JSN* joint space narrowing, *ACPA* anti-citrullinated protein antibody, *Fib* fibrinogen, *cit* citrullinated, *Vim* vimentin, *H* histone, *CEP-1* citrullinated enolase peptide-1, *Fil* filaggrin; numbers referring to amino acid sequence, *NA* not applicable

^a^Mean (SD)

^b^Number (percentage)

^c^Median [25, 75 percentile]

### ACPA reactivities and disease activity at baseline

The DAS, SJC44 and ultrasound grey scale and power Doppler scores were significantly higher in patients who were negative for anti-CCP. Similarly, lack of the most commonly occurring ACPA reactivities (Fibβ 60–74, Vim 60–75 and H4 31–50) was associated with higher DAS, SJC44 and ultrasound grey scale and power Doppler to a statistically significant level (Table 2). When performing the same analyses on the anti-CCP positive cohort only, there were no differences in DAS, SJC44 or ultrasound scores between the patients testing positive versus negative for the ACPA reactivities (Additional file 1: Table S1). The DAS, SJC44 and ultrasound grey scale and power Doppler scores were negatively correlated with the majority of the ACPA reactivity levels at baseline (Additional file 1: Table S2). This association was not seen when considering the anti-CCP-positive cohort only (Additional file 1: Table S3). Number of ACPA reactivities was not correlated with ESR and CRP (data not shown).

**Table 2:** Baseline disease characteristics (median values) in anti-CCP and ACPA reactivity-positive versus reactivity-negative patients

		ESR		CRP		DAS		SJC44		RAI		US GS		US PD		vdHSS E		vdHSS JSN		vdHSS T	
			*p*		*p*		*p*		*p*		*p*		*p*		*p*		*p*		*p*		*p*
Anti-CCP	+ (82%)	21	0.10	7	0.20	3.24	*< 0.01*	8	*< 0.01*	7	0.17	16	*< 0.01*	6	*< 0.01*	3	0.72	1	0.10	4	0.37
	- (18%)	13		7		4.09		18		8		33		14		3		1.5		4.5	
Fibβ 60–74	+ (75%)	20	0.63	7	*0.04*	3.28	*0.02*	7	*< 0.01*	7	0.65	17	*< 0.01*	6	*< 0.01*	3	0.97	1	0.71	4.5	0.73
	- (25%)	19		10		3.87		14		7		28		11		3		0.5		3.5	
Vim 60–75	+ (73%)	18	0.95	7	0.54	3.29	*0.05*	8	*< 0.01*	6	0.83	17	*< 0.01*	6	*0.02*	3	0.96	1	0.26	4.5	0.95
	- (27%)	21		7		3.77		14		7		23		9		3		1		4	
H4 31–50	+ (67%)	21	0.39	8	0.65	3.27	*0.01*	8	*< 0.01*	6	*0.03*	17	*0.01*	6	*0.01*	3	0.88	1	0.94	4.5	0.70
	- (33%)	17		7		3.84		13		10		21		9		3		0.75		3.25	
CEP-1	+ (65%)	21	0.13	8	0.93	3.32	0.35	9	0.06	7	0.47	17	*0.03*	6	0.12	3	0.76	1	0.46	4.5	0.54
	- (35%)	17		6		3.36		11		7		21		7		3		0.5		3.5	
F*il* 307–324	+ (63%)	21	0.15	8	0.70	3.29	0.06	7	*< 0.01*	7	0.54	17	*0.01*	6	*< 0.01*	3	0.94	1	0.98	4	0.91
	- (37%)	17		7		3.64		13		7		21		9		3		1		4	
Fibα 573	+ (57%)	22	0.13	8	0.85	3.27	*0.03*	7	*< 0.01*	6	0.14	17	*0.05*	6	0.12	3	0.61	1	0.23	4.5	0.37
	- (43%)	16		7		3.42		12		8		21		8		3		0.5		4	
Fibβ 36–52	+ (54%)	21	0.08	8	0.78	3.26	*0.05*	8	*< 0.01*	6	0.06	17	0.06	6	*0.03*	3	0.58	1	*0.05*	4.5	0.19
	- (46%)	17		7		3.63		11		8		19		8		3		0.5		3.5	
H3 1–30	+ (49%)	18	0.97	7	0.40	3.29	0.48	8	0.09	6	1.00	17	*0.04*	6	0.09	3	0.62	0.5	*0.04*	4	0.16
	- (51%)	20		7		3.37		10		7		19		7		3		1		4.5	
H4 14–34	+ (48%)	21	0.13	8	0.74	3.27	0.28	7	*< 0.01*	7	0.77	17	0.30	7	0.86	3	0.39	1	0.30	4	0.28
	- (52%)	18		7		3.45		11		7		20		6		3		1		4.5	
H3 21–44	+ (44%)	22	0.09	9	0.21	3.31	0.69	9	0.07	7	0.84	17	0.28	6	0.41	3	0.68	0.5	0.14	4	0.32
	- (56%)	18		6		3.36		10		7		19		7		3		1		4.5	
Fibα 621–635	+ (43%)	22	0.36	8	0.84	3.29	0.09	7	*< 0.01*	7	0.54	16	*0.02*	6	0.07	2.5	0.46	0.5	0.30	4	0.34
	- (57%)	18		7		3.37		12		7		20		7		3		1		4.5	
Vim 2–17	+ (41%)	19	0.72	7	0.49	3.29	0.34	7	*< 0.01*	7	0.48	17	0.08	6	0.13	3	0.50	1	0.61	4	0.45
	- (59%)	20		7		3.36		11		7		20		7		3		1		4	
Fibα 36–50	+ (36%)	20	0.73	8	0.86	3.29	0.68	8	*0.04*	7	0.46	17	0.08	6	*0.05*	3	0.84	0.5	0.29	4	0.59
	- (64%)	19		7		3.41		11		7		19		7		3		1		4	
Fibα 591	+ (32%)	19	0.70	6	0.29	3.29	*0.05*	7	*< 0.01*	6	0.28	17	0.53	6	0.88	3	0.86	1	0.33	4.5	0.70
	- (68%)	20		8		3.40		11		7		19		7		3		0.5		4	
Fibβ 74	+ (30%)	22	0.19	8	0.87	3.25	0.53	9	0.12	8	0.74	17	0.08	6	0.67	2.5	0.57	0.5	0.32	4	0.48
	- (70%)	18		7		3.33		10		7		19		7		3		1		4	
Fibβ 72	+ (12%)	21	0.25	5	0.59	3.59	0.37	10	0.48	9	0.52	22	0.19	9	0.06	3	0.73	0.5	0.72	4	0.98
	- (88%)	19		7		3.31		9		7		17		6		3		1		4	

Anti-citrullinated protein antibody (ACPA) reactivities are sorted by decreasing frequency in the cohort: *p* values (*p*) were derived from the Mann-Whitney U test; statistically significant differences are in italics

*Abbreviations*: *ESR* erythrocyte sedimentation rate (millimetre/hour, 1–140), *CRP* C-reactive protein (milligram/litre), *DAS* Disease Activity Score (0–10), *SJC* swollen joint count (0–44), *RAI* Ritchie articular index (0–78), *US* ultrasound, *GS* grey scale (0–96), *PD* power Doppler (0–96), *vdHSS* van der Heijde modified Sharp score, *E* erosion (0–280), *JSN* joint space narrowing (0–168), *T* total (0–448), *anti-CCP* anti-cyclic citrullinated peptide, *Fib* fibrinogen, *Vim* vimentin, *H* histone, *CEP-1* citrullinated enolase peptide-1, *Fil* filaggrin; numbers referring to amino acid sequence

### ACPA reactivities and disease activity after initiation of DMARD treatment

**Fig. 2:**
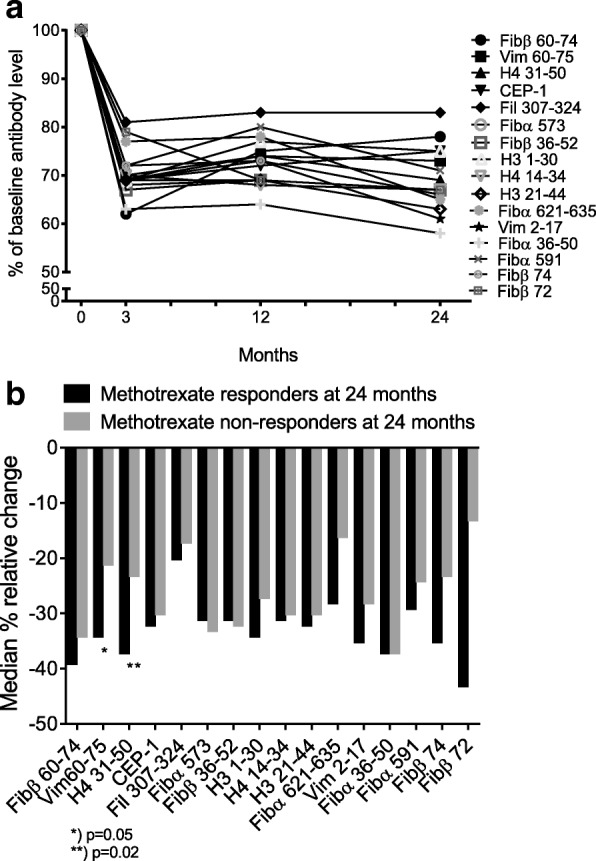
Relative change in levels of various anti-citrullinated protein antibody (ACPA) reactivities in patients with early rheumatoid arthritis (RA) (only baseline seropositive patients included) **a** Relative change between baseline and 3, 12 and 24 months, **b** Relative change after 3 months, comparing patients on methotrexate monotherapy at 24 months (*n* = 113) to patients on triple and/or biological disease-modifying antirheumatic drugs (bDMARDs) at 24 months (*n* = 82). Fib, fibrinogen; Vim, vimentin; H, histone; CEP-1, citrullinated enolase peptide-1; Fil, Filaggrin, numbers referring to amino acid sequence

ACPA reactivity levels declined significantly after initiation of DMARD treatment (Fig. 2a) with the most prominent drop in ACPA reactivity levels occuring within the first 3 months. The delta median change in DAS (after 6, 12 and 24 months), ultrasound grey scale and power Doppler (both after 12 and 24 months) was higher in the ACPA reactivity negative patients, with few exceptions (Table 3). However, these differences between the ACPA reactivity negative versus positive patients in DAS, ultrasound grey scale and power Doppler evened out during follow up (data not shown). The most pronounced relative change comparing baseline levels to levels after 3 months was seen for Fibβ 60–74 and the least marked decrease was for Fil 307–324 (38% vs. 19% decrease, both *p* < 0.001; Fig. 2a). We wanted to investigate whether the relative change in ACPA reactivity levels after 3 months differed between patients with lasting methotrexate treatment response and patients requiring triple treatment and/or bDMARDs over the 2 years of follow up. A greater relative change after 3 months of DMARD treatment was seen in most ACPA reactivities in patients who were methotrexate monotherapy responders, and to a statistically significant degree for Vim 60–75 and H4 31–50 (Fig. 2b).

**Table 3:** Change in disease characteristics (delta median values) in ACPA reactivity-positive versus reactivity-negative patients

		Delta DAS 6 months		Delta DAS 12 months		Delta DAS 24 months		Delta US GS 12 months		Delta US GS 24 months		Delta US PD 12 months		Delta US PD 24 months		Delta vdHSS T 12 months		Delta vdHSS T 24 months	
			*p*		*p*		*p*		*p*		*p*		*p*		*p*		*p*		*p*
Fibβ 60–74	+	−1.9	*0.04*	−2.0	*< 0.01*	− 2. 0	*0.02*	− 12	*< 0.01*	− 13	*< 0.01*	−6	*< 0.01*	− 6	*< 0.01*	0.5	0.92	1.0	0.69
	–	− 2.5		−2.6		−2.6		− 19		−21		−9		−10		0.5		1.0	
Vim 60–75	+	−1.9	*< 0.01*	− 1.9	*< 0.01*	− 2.0	*0.02*	− 12	*< 0.01*	− 13	*< 0.01*	− 6	0.06	− 6	*0.02*	0.5	0.59	1.0	0.68
	–	−2.6		− 2.6		− 2.6		−17		− 17		−8		−8		0.5		1.0	
H4 31–50	+	−1.9	*< 0.01*	− 1.9	*< 0.01*	−2.0	*< 0.01*	− 12	*0.05*	− 13	*< 0.01*	− 6	0.08	− 6	*0.02*	0.5	0.15	1.0	0.81
	–	−2.5		− 2.8		− 2.7		− 15		−17		−7		−8		0.5		1.0	
CEP-1	+	−2.0	0.75	− 2.1	0.79	−2.1	0.48	−12	0.23	− 14	0.24	−6	0.17	−6	0.14	0.5	0.50	1.0	0.41
	–	−2.1		−2.2		−2.0		−14		−14		−7		− 7		0.5		1.0	
Fil 307–324	+	−1.9	*0.01*	−1.8	*< 0.01*	− 1.9	*0.01*	− 12	0.06	− 13	*0.01*	−6	*0.03*	−5	*0.01*	0.5	0.75	1.0	0.76
	–	−2.4		−2.5		−2.5		−16		−16		−7		− 7		0.5		1.0	
Fibα 573	+	−1.9	*< 0.01*	−1.8	*< 0.01*	− 1.9	*< 0.01*	− 13	0.36	− 14	0.12	−6	0.21	−6	0.08	0.5	0.89	1.0	0.77
	–	−2.5		−2.6		−2.4		−13		−15		−7		−7		0.5		1.5	
Fibβ 36–52	+	−1.9	*0.02*	−1.9	*< 0.01*	− 1.9	*< 0.01*	−12	0.23	−14	0.09	−6	0.08	−5	*0.02*	0.5	0.89	1.0	0.58
	–	−2.4		−2.5		−2.5		−14		− 14		−7		−7		0.5		1.5	
H3 1–30	+	−2.1	0.97	− 2.2	0.97	− 2.0	0.44	−12	0.31	− 13	*0.04*	−6	0.25	−6	0.09	0.5	0.24	1.0	0.45
	–	−2.0		−2.1		−2.2		−14		−16		−7		−7		0.5		1.2	
H4 14–34	+	−1.9	0.07	−2.0	0.12	−2.0	0.14	−12	0.46	−12	*0.02*	−7	0.63	−6	0.54	0.5	0.32	1.5	0.10
	–	−2.1		−2.3		−2.2		−14		−16		−6		−6		0.5		1.0	
H3 21–44	+	−2.0	0.48	−2.0	0.31	−2.0	0.37	−12	0.29	−13	0.39	−6	0.28	−6	0.38	0.5	0.39	1.5	0.76
	–	−2.0		−2.2		−2.2		−15		−15		−7		−7		0.5		1.0	
Fibα 621–635	+	−1.7	*0.04*	−1.8	*0.04*	−1.9	*0.03*	−11	*0.02*	−13	*0.01*	−6	0.09	−6	*0.05*	0.5	0.20	1.0	0.22
	–	−2.3		−2.4		−2.2		−15		−16		−7		−7		0.5		1.5	
Vim 2–17	+	−1.9	*0.04*	−1.8	*0.02*	−1.8	*0.01*	−12	0.12	−12	*< 0.01*	−6	0.21	−5	*0.04*	0.5	0.90	1.0	0.87
	–	−2.2		−2.3		−2.3		−14		−16		−7		−7		0.5		1.0	
Fibα 36–50	+	−2.0	0.83	− 2.0	0.74	− 2.0	0.45	−11	0.09	− 12	0.10	−5	*0.02*	−5	*0.02*	0.5	0.61	1.0	0.52
	–	−2.0		−2.2		−2.2		−14		−15		−7		−7		0.5		1.0	
Fibα 591	+	−1.9	0.08	− 1.8	*0.03*	− 1.8	*0.01*	− 13	0.69	− 16	0.70	−6	0.84	−6	0.77	0.5	0.41	1.0	0.58
	–	−2.2		−2.3		−2.3		−13		−14		−6		−7		0.5		1.5	
Fibβ 74	+	−1.8	0.41	−2.0	0.79	−2.1	0.55	−12	0.36	−13	0.94	−7	0.82	−7	0.98	0.5	0.06	1.5	0.54
	–	−2.1		−2.2.		−2.0		−13		−14		−6		−6		0.5		1.0	
Fibβ 72	+	−2.0	0.84	−2.0	0.73	−1.9	0.78	− 16	0.19	− 17	0.21	−7	0.13	−9	0.11	0.2	0.98	0.5	0.51
	–	−2.0		−2.2.		−2.1		−12		−14		−6		−6		0.5		1.0	

Anti-citrullinated protein antibody (ACPA) reactivities are sorted by decreasing frequency in the cohort: *p* values (*p*) were derived from the Mann-Whitney U test; statistically significant differences are in italics

*Abbreviations*: *DAS* Disease Activity Score (0–10), *SJC* swollen joint count (0–44), *US* ultrasound, *GS* grey scale (0–96), *PD* power Doppler 0–96), *vdHSS* van der Heijde modified Sharp score, *T* total (0–448), *Fib* fibrinogen, *Vim* vimentin, *H* histone, *CEP-1* citrullinated enolase peptide-1, *Fil* filaggrin; numbers referring to amino acid sequence

### ACPA reactivities at baseline and prediction of treatment response

There was no difference in median baseline level of the individual ACPA reactivities (data not shown) or baseline median number of ACPA reactivities between patients with successful methotrexate monotherapy according to EULAR good/moderate response and patients with no treatment response after 3 months (9 [4, 12] vs. 10 [4, 11]; *p* = 0.80). Likewise, no difference was seen in median baseline level of the individual ACPA reactivities (data not shown) or median number of ACPA reactivities in patients reaching remission vs. not reaching remission according to DAS (8 [3, 12] vs. 10 [6, 12]; *p* = 0.16) or ACR/EULAR Boolean criteria (9 [4, 12] vs. 9 [5, 11]; *p* = 0.74) after 6 months of methotrexate treatment. Median baseline number of ACPA reactivities for patients reaching vs. not reaching DAS remission at 12 months was 8 [4, 11] vs. 10 [6, 11], *p* = 0.26 and for 24 months 8 [4, 12] vs. 10 [4, 11], *p* = 0.87. For ACR EULAR Boolean remission at 12 months, the corresponding numbers were 7 [3, 11] vs. 10 [5, 11], *p* = 0.08 and at 24 months 8 [3, 11] vs. 9 [6, 12], *p* = 0.36, respectively.

When stratifying by number of ACPA reactivities, there was a trend that patients with no ACPA reactivities present at baseline were more likely to be in methotrexate monotherapy remission according to DAS after 6 months than patients with 1-5, 6-8or ≥9 ACPA reactivities present at baseline (70% vs. 53%, 53%, and 47%, respectively; *p* = 0.18). No such trend was found for DAS remission at 12 months (81% vs. 67%, 77% and 67%, respectively; *p* = 0.50) or 24 months (89% vs. 85%, 78%, and 78%, respectively; *p* = 0.71). When comparing the proportion of patients in remission according to the ACR/EULAR Boolean remission criteria in relation to number of baseline ACPA reactivities, no trend was seen at 6, 12 or 24 months (data not shown).

### ACPA and radiographic damage

**Fig. 3:**
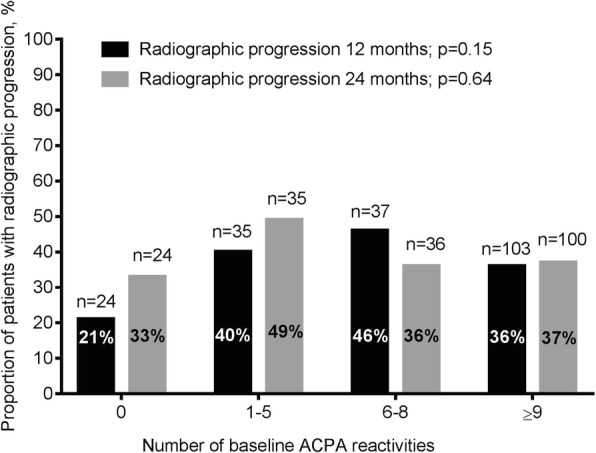
Proportion of patients with radiographic progression by number of baseline ACPA reactivities after 12 months (*n* = 199) and 24 months (*n* = 195)

Anti-CCP positivity was not associated with baseline radiographic scores, and neither of the individual ACPA reactivity levels was associated with baseline radiographic scores or baseline erosive disease (Table 2). Overall median observed change in van der Heijde modified Sharp total score after 12 and 24 months did not differ between ACPA reactivity-positive vs. reactivity-negative patients (Table 3). Baseline presence and/or levels of any of the ACPA reactivities were not associated with progression of radiographic damage or change in radiographic score after 12 and 24 months, and seroreversion of any ACPA reactivity after 3 months of treatment was not associated with less radiographic progression after 12 and 24 months (data not shown). Outcomes did not differ when performing subanalyses on the anti-CCP-positive cohort only. Number of ACPA reactivities was not associated with baseline radiographic scores or presence of baseline erosive disease (Table 4). However, there was a trend towards less radiographic progression after 12 and 24 months in patients with no baseline ACPA reactivities compared with 1–5, 6–8 and ≥ 9 reactivities (Fig. 3), and also when comparing no baseline ACPA reactivities with ≥ 1 reactivities (21% vs. 39% at 12 months, *p* = 0.10 and 33% vs. 39% at 24 months, *p* = 0.66).

**Table 4:** Association between number of baseline ACPA reactivities and van der Heijde modified Sharp scores (erosion, joint space narrowing and total) and association between number of baseline ACPA reactivities and baseline erosive disease

	Number of ACPA reactivities				
	0 *n* = 27	1–5 n = 39	6–8 *n* = 37	≥ 9 *n* = 114	*p* value
vdHSS erosion^a^	3.0 [1.0, 5.8]	3.0 [1.5, 4.0]	3.0 [2.0, 4.0]	3.0 [1.0, 4.5]	0.87
vdHSS joint space narrowing^a^	1.5 [0.0, 4.8]	0.5 [0.0, 2.0]	0.5 [0.0, 3.0]	1.0 [0.0, 3.8]	0.54
vdHSS total^a^	3.0 [1.5, 11.2]	3.5 [1.5, 6.0]	4.5 [2.5, 7.0]	4.2 [1.5, 9.0]	0.83
Erosive disease^b^	15 (56)	21 (54)	21 (57)	60 (53)	0.97

*Abbreviations:* *ACPA* Anti-citrullinated protein antibody, *vdHSS* van der Heijde modified Sharp score

^a^Median [25,75 percentile]

^b^Number (percentage)

## Discussion

To our knowledge, this is the first study to examine the relationship between ACPA reactivities in patients with early RA classified according to the 2010 ACR/EULAR criteria and imaging and serological and clinical disease activity measures, and their potential use in prediction of treatment response and radiographic progression [42]. The prevalence of ACPA reactivities was associated with anti-CCP/RF positivity, and baseline disease activity measures and ultrasound scores were lower in ACPA reactivity-positive compared to ACPA reactivity-negative patients. ACPA reactivity levels decreased after initiation of DMARD treatment, but the clinical implications of measuring ACPA reactivities to predict treatment response and radiographic progression in this cohort of intensively treated patients with early RA were limited.

High levels of anti-CCP antibodies have previously been shown to be a risk factor for erosive disease [1, 2], and selected ACPA reactivities have also been shown to be associated with osteoclast activation and radiographic damage [23, 24, 26, 27, 43]. Kastbom et al. have shown that ACPA reactivity levels declined after 3 months of methotrexate treatment, and that disappearance of certain ACPA reactivities was associated with less 2-year radiographic progression [23]. In our study, levels of all ACPA reactivities declined after 3 months of DMARD treatment. None of the individual ACPA reactivities analysed were associated with baseline erosive disease or radiographic progression, nor was seroreversion of individual ACPA reactivities associated with less radiographic progression over the 2 years of follow up in our study. Presence, but not titre, of reactivities to citrullinated fibrinogen was associated with faster joint destruction in the ESPOIR cohort of patients with very early RA fulfilling either the 1987 ACR criteria at inclusion or the 2010 ACR/EULAR criteria within 3 years of inclusion [26]. We identified a trend that patients with ≥ 1 ACPA reactivity at baseline experienced more radiographic progression at 12 and 24 months compared to patients with no ACPA reactivities present, but the findings were not significant. Lack of significant association between number of ACPA reactivities and radiographic progression has previously also been described in the Leiden early arthritis cohort where patients fulfilled the 1987 ACR criteria within 1 year after inclusion [29–31]. Previous studies have not demonstrated association between the individual ACPA reactivities and disease activity measures in early undifferentiated arthritis and early RA [29–32]. ACPA reactivities present in anti-CCP-negative patients with RA have not been shown to be associated with clinical or prognostic parameters [22]. As previously published, seropositive patients in the ARCTIC cohort had significantly lower disease activity compared to seronegative patients [44], similar to what has been reported in other cohorts [45, 46]. The most prevalent ACPA reactivities in our study were negatively correlated with the DAS, SJC44 and ultrasound grey scale and power Doppler scores at baseline, and the disease activity measurements were numerically higher in patients who were negative for the individual ACPA reactivities, which has not been described previously. These differences were no longer present when excluding the anti-CCP-negative subgroup and are thus potentially a consequence of the 2010 ACR/EULAR classification criteria, where the anti-CCP/RF-negative patients require greater joint involvement than the seropositive patients to fulfill the criteria [42, 44].

Current guidelines for treat-to-target emphasise the importance of improvement in disease activity within 3 months (EULAR good/moderate response) and attainment of the treatment target (ACR/EULAR Boolean remission) within 6 months after initiating treatment [47]. A greater decrease in ACPA reactivity levels has been demonstrated in treatment responders compared to non-responders [23]. In our cohort we identified a similar trend whereby reduction in ACPA reactivity levels after 3 months was more pronounced in patients remaining on methotrexate monotherapy after 2 years of follow up, but we did not observe any baseline predictors of treatment response.

Comparison between our results and those of other studies may be limited by the fact that patients in most previous studies were classified by the 1987 ACR criteria or in cohorts of patients treated less intensively. The relatively few anti-CCP-negative patients in comparison with the larger amount of anti-CCP positive patients reflects an effect of implementing the 2010 ACR/EULAR classification criteria [33] and in this study, the number of patients in the subgroups of anti-CCP and ACPA reactivity-negative patients is somewhat small for meaningful comparisons. When applying the 2010 ACR/EULAR criteria as inclusion criteria, the seronegative patients included in the study were required to have more joint involvement with > 10 clinically involved joints [42]. Patients with a clinical seronegative RA diagnosis involving fewer joints were not included. Strengths of the study were that all patients were classified by the 2010 ACR/EULAR criteria, were DMARD and corticosteroid naïve at inclusion and were treated according to a standardised treatment protocol adhering to current treatment recommendations [33]. The treatment regimen of the ARCTIC trial is well in line with current EULAR recommendations for treatment of early RA [47]. Such intensive treatment may suppress the RA disease activity to such a degree that prognostic markers identified in previous less strictly controlled RA cohorts may no longer be present or of clinical relevance. The current study was also strengthened by a relatively large sample size and a broad collection of serological, clinical and imaging data.

## Conclusions

New classification criteria and modern intensive treatment strategies have altered the disease course of early RA, and consequently established predictors for treatment response and progression of radiographic damage should be re-evaluated in appropriate cohorts. In this inception cohort of RA patients classified according to the ACR/EULAR 2010 criteria, the prevalence of ACPA reactivities differed in subgroups according to anti-CCP and RF status, and ACPA reactivity levels decreased after initiation of DMARD treatment. There were no apparent associations with disease activity, prediction of treatment response or radiographic progression, and further studies in larger patient samples are needed to understand the role of ACPA reactivities in patients with RA classified according to the 2010 ACR/EULAR criteria.

## Additional file

Additional file 1: **Table S1.** Disease characteristics (median values) in ACPA reactivity-positive versus reactivity-negative patients in the anti-CCP-positive sub-group (*n* = 178); *p* values were derived from the Mann-Whitney U test and statistically significant differences are marked in bold. Reactivities are sorted by decreasing frequency in the cohort. **Table S2.** Correlation between ACPA reactivities and disease activity (baseline). Spearman correlation (*p* value). **Table S3.** Correlation between reactivities and disease activity in anti-CCP-positive patients (baseline). Spearman correlation (*p* value). (DOCX 41 kb)

## Abbreviations

ACPA: Anti-citrullinated protein antibody
ACR: American College of Rheumatology
Anti-CCP: Anti-cyclic citrullinated peptide
ARCTIC: Aiming for remission in rheumatoid arthritis: a randomized trial examining the benefit of ultrasound in a clinical tight control regimen
AU: Accredited units
bDMARDs: Biologic disease-modifying antirheumatic drug
CEP-1: Citrullinated enolase peptide-1
CRP: C-reactive protein
DAS: Disease Activity Score
DMARD: Disease-modifying antirheumatic drug
E: Erosion
ELISA: Enzyme-linked immunosorbent assay
ESR: Erythrocyte sedimentation rate
EULAR: European League Against Rheumatism
FEIA: Fluorometric enzyme immunocapture assay
Fib: Fibrinogen
Fil: Filaggrin
H: Histone
JSN: Joint space narrowing
mL: Milliliter
RA: Rheumatoid arthritis
RAI: Ritchie Articular Index
RF: Rheumatoid factor
SJC: Swollen joint count
T: Total
VAS: Visual analogue scale
vdHSS: van der Heijde modified Sharp score
Vim: Vimentin

## References

[1] Berglin E, Johansson T, Sundin U, Jidell E, Wadell G, Hallmans G (2006). Radiological outcome in rheumatoid arthritis is predicted by presence of antibodies against cyclic citrullinated peptide before and at disease onset, and by IgA-RF at disease onset. Ann Rheum Dis.

[2] Syversen SW, Gaarder PI, Goll GL, Odegard S, Haavardsholm EA, Mowinckel P (2008). High anti-cyclic citrullinated peptide levels and an algorithm of four variables predict radiographic progression in patients with rheumatoid arthritis: results from a 10-year longitudinal study. Ann Rheum Dis.

[3] Forslind K, Ahlmen M, Eberhardt K, Hafstrom I, Svensson B, Group BS (2004). Prediction of radiological outcome in early rheumatoid arthritis in clinical practice: role of antibodies to citrullinated peptides (anti-CCP). Ann Rheum Dis.

[4] Lindqvist E, Eberhardt K, Bendtzen K, Heinegard D, Saxne T (2005). Prognostic laboratory markers of joint damage in rheumatoid arthritis. Ann Rheum Dis.

[5] Nielen MM, van der Horst AR, van Schaardenburg D, van der Horst-Bruinsma IE, van de Stadt RJ, Aarden L (2005). Antibodies to citrullinated human fibrinogen (ACF) have diagnostic and prognostic value in early arthritis. Ann Rheum Dis.

[6] Ronnelid J, Wick MC, Lampa J, Lindblad S, Nordmark B, Klareskog L (2005). Longitudinal analysis of citrullinated protein/peptide antibodies (anti-CP) during 5 year follow up in early rheumatoid arthritis: anti-CP status predicts worse disease activity and greater radiological progression. Ann Rheum Dis.

[7] Machold KP, Stamm TA, Nell VP, Pflugbeil S, Aletaha D, Steiner G (2007). Very recent onset rheumatoid arthritis: clinical and serological patient characteristics associated with radiographic progression over the first years of disease. Rheumatology (Oxford).

[8] Pruijn GJ, Wiik A, van Venrooij WJ (2010). The use of citrullinated peptides and proteins for the diagnosis of rheumatoid arthritis. Arthritis Res Ther.

[9] Schellekens GA, de Jong BA, van den Hoogen FH, van de Putte LB, van Venrooij WJ (1998). Citrulline is an essential constituent of antigenic determinants recognized by rheumatoid arthritis-specific autoantibodies. J Clin Invest.

[10] Girbal-Neuhauser E, Durieux JJ, Arnaud M, Dalbon P, Sebbag M, Vincent C (1999). The epitopes targeted by the rheumatoid arthritis-associated antifilaggrin autoantibodies are posttranslationally generated on various sites of (pro)filaggrin by deimination of arginine residues. J Immunol.

[11] Snir O, Widhe M, von Spee C, Lindberg J, Padyukov L, Lundberg K (2009). Multiple antibody reactivities to citrullinated antigens in sera from patients with rheumatoid arthritis: association with HLA-DRB1 alleles. Ann Rheum Dis.

[12] van der Woude D, Rantapaa-Dahlqvist S, Ioan-Facsinay A, Onnekink C, Schwarte CM, Verpoort KN (2010). Epitope spreading of the anti-citrullinated protein antibody response occurs before disease onset and is associated with the disease course of early arthritis. Ann Rheum Dis.

[13] van de Stadt LA, van der Horst AR, de Koning MH, Bos WH, Wolbink GJ, van de Stadt RJ (2011). The extent of the anti-citrullinated protein antibody repertoire is associated with arthritis development in patients with seropositive arthralgia. Ann Rheum Dis.

[14] Lundberg K, Bengtsson C, Kharlamova N, Reed E, Jiang X, Kallberg H (2013). Genetic and environmental determinants for disease risk in subsets of rheumatoid arthritis defined by the anticitrullinated protein/peptide antibody fine specificity profile. Ann Rheum Dis.

[15] Ronnelid J, Hansson M, Mathsson-Alm L, Cornillet M, Reed E, Jakobsson PJ (2018). Anticitrullinated protein/peptide antibody multiplexing defines an extended group of ACPA-positive rheumatoid arthritis patients with distinct genetic and environmental determinants. Ann Rheum Dis.

[16] Sokolove J, Bromberg R, Deane KD, Lahey LJ, Derber LA, Chandra PE (2012). Autoantibody epitope spreading in the pre-clinical phase predicts progression to rheumatoid arthritis. PLoS One.

[17] Brink M, Hansson M, Mathsson L, Jakobsson PJ, Holmdahl R, Hallmans G (2013). Multiplex analyses of antibodies against citrullinated peptides in individuals prior to development of rheumatoid arthritis. Arthritis Rheum.

[18] Johansson L, Pratesi F, Brink M, Arlestig L, D'Amato C, Bartaloni D (2016). Antibodies directed against endogenous and exogenous citrullinated antigens pre-date the onset of rheumatoid arthritis. Arthritis Res Ther..

[19] Arkema EV, Goldstein BL, Robinson W, Sokolove J, Wagner CA, Malspeis S (2013). Anti-citrullinated peptide autoantibodies, human leukocyte antigen shared epitope and risk of future rheumatoid arthritis: a nested case-control study. Arthritis Res Ther.

[20] Hansson M, Mathsson L, Schlederer T, Israelsson L, Matsson P, Nogueira L (2012). Validation of a multiplex chip-based assay for the detection of autoantibodies against citrullinated peptides. Arthritis Res Ther..

[21] Wagner CA, Sokolove J, Lahey LJ, Bengtsson C, Saevarsdottir S, Alfredsson L (2015). Identification of anticitrullinated protein antibody reactivities in a subset of anti-CCP-negative rheumatoid arthritis: association with cigarette smoking and HLA-DRB1 ‘shared epitope’ alleles. Ann Rheum Dis.

[22] van Heemst J, Trouw LA, Nogueira L, van Steenbergen HW, van der Helm-van Mil AH, Allaart CF (2015). An investigation of the added value of an ACPA multiplex assay in an early rheumatoid arthritis setting. Arthritis Res Ther..

[23] Kastbom A, Forslind K, Ernestam S, Geborek P, Karlsson JA, Petersson IF (2016). Changes in the anticitrullinated peptide antibody response in relation to therapeutic outcome in early rheumatoid arthritis: results from the SWEFOT trial. Ann Rheum Dis.

[24] Hensvold AH, Joshua V, Li W, Larkin M, Qureshi F, Israelsson L (2015). Serum RANKL levels associate with anti-citrullinated protein antibodies in early untreated rheumatoid arthritis and are modulated following methotrexate. Arthritis Res Ther..

[25] Figueiredo CP, Bang H, Cobra JF, Englbrecht M, Hueber AJ, Haschka J (2017). Antimodified protein antibody response pattern influences the risk for disease relapse in patients with rheumatoid arthritis tapering disease modifying antirheumatic drugs. Ann Rheum Dis.

[26] Cornillet M, Ajana S, Ruyssen-Witrand A, Constantin A, Degboe Y, Cantagrel A et al. Autoantibodies to human citrullinated fibrinogen and their subfamilies to the alpha36-50Cit and beta60-74Cit fibrin peptides similarly predict radiographic damages: a prospective study in the French ESPOIR cohort of very early arthritides. Rheum. 2016;55:1859-70. 10.1093/rheumatology/kew014 26961744

[27] Harre U, Georgess D, Bang H, Bozec A, Axmann R, Ossipova E (2012). Induction of osteoclastogenesis and bone loss by human autoantibodies against citrullinated vimentin. J Clin Invest.

[28] Montes A, Perez-Pampin E, Calaza M, Gomez-Reino JJ, Gonzalez A (2012). Association of anti-citrullinated vimentin and anti-citrullinated alpha-enolase antibodies with subsets of rheumatoid arthritis. Arthritis Rheum.

[29] Willemze A, Bohringer S, Knevel R, Levarht EW, Stoeken-Rijsbergen G, Houwing-Duistermaat JJ (2012). The ACPA recognition profile and subgrouping of ACPA-positive RA patients. Ann Rheum Dis.

[30] van Beers JJ, Willemze A, Jansen JJ, Engbers GH, Salden M, Raats J (2013). ACPA fine-specificity profiles in early rheumatoid arthritis patients do not correlate with clinical features at baseline or with disease progression. Arthritis Res Ther..

[31] Scherer HU, van der Woude D, Willemze A, Trouw LA, Knevel R, Syversen SW (2011). Distinct ACPA fine specificities, formed under the influence of HLA shared epitope alleles, have no effect on radiographic joint damage in rheumatoid arthritis. Ann Rheum Dis.

[32] Fisher BA, Plant D, Brode M, van Vollenhoven RF, Mathsson L, Symmons D (2011). Antibodies to citrullinated alpha-enolase peptide 1 and clinical and radiological outcomes in rheumatoid arthritis. Ann Rheum Dis.

[33] Haavardsholm EA, Aga AB, Olsen IC, Lillegraven S, Hammer HB, Uhlig T (2016). Ultrasound in management of rheumatoid arthritis: ARCTIC randomised controlled strategy trial. BMJ.

[34] van der Heijde DM, van 't Hof MA, van Riel PL, Theunisse LA, Lubberts EW, van Leeuwen MA (1990). Judging disease activity in clinical practice in rheumatoid arthritis: first step in the development of a disease activity score. Ann Rheum Dis.

[35] Ritchie DM, Boyle JA, McInnes JM, Jasani MK, Dalakos TG, Grieveson P (1968). Clinical studies with an articular index for the assessment of joint tenderness in patients with rheumatoid arthritis. Q J Med.

[36] van Gestel AM, Prevoo ML, van 't Hof MA, van Rijswijk MH, van de Putte LB, van Riel PL (1996). Development and validation of the European league against rheumatism response criteria for rheumatoid arthritis. Comparison with the preliminary American College of Rheumatology and the World Health Organization/international league against rheumatism criteria. Arthritis Rheum.

[37] Felson DT, Smolen JS, Wells G, Zhang B, van Tuyl LH, Funovits J (2011). American College of Rheumatology/European league against rheumatism provisional definition of remission in rheumatoid arthritis for clinical trials. Ann Rheum Dis.

[38] van der Heijde D (1999). How to read radiographs according to the sharp/van der Heijde method. J Rheumatol.

[39] van der Heijde D, van der Helm-van Mil AH, Aletaha D, Bingham CO, Burmester GR, Dougados M (2013). EULAR definition of erosive disease in light of the 2010 ACR/EULAR rheumatoid arthritis classification criteria. Ann Rheum Dis.

[40] Hammer HB, Bolton-King P, Bakkeheim V, Berg TH, Sundt E, Kongtorp AK (2011). Examination of intra and interrater reliability with a new ultrasonographic reference atlas for scoring of synovitis in patients with rheumatoid arthritis. Ann Rheum Dis.

[41] Swinscow TDV. Statistics at square one, ninth edn. London: BMJ publishing Group; 1997.

[42] Aletaha D, Neogi T, Silman AJ, Funovits J, Felson DT, Bingham CO (2010). 2010 Rheumatoid arthritis classification criteria: an American College of Rheumatology/European league against rheumatism collaborative initiative. Arthritis Rheum.

[43] Krishnamurthy A, Joshua V, Haj Hensvold A, Jin T, Sun M, Vivar N (2016). Identification of a novel chemokine-dependent molecular mechanism underlying rheumatoid arthritis-associated autoantibody-mediated bone loss. Ann Rheum Dis.

[44] Nordberg LB, Lillegraven S, Lie E, Aga AB, Olsen IC, Hammer HB et al. Patients with seronegative RA have more inflammatory activity compared with patients with seropositive RA in an inception cohort of DMARD-naive patients classified according to the 2010 ACR/EULAR criteria. Ann Rheum Dis. 2016;76:341-5. 10.1136/annrheumdis-2015-208873 27094444

[45] Barra L, Pope JE, Orav JE, Boire G, Haraoui B, Hitchon C (2014). Prognosis of seronegative patients in a large prospective cohort of patients with early inflammatory arthritis. J Rheumatol.

[46] Choi ST, Lee KH (2018). Clinical management of seronegative and seropositive rheumatoid arthritis: a comparative study. PLoS One.

[47] Smolen JS, Landewe R, Bijlsma J, Burmester G, Chatzidionysiou K, Dougados M et al. EULAR recommendations for the management of rheumatoid arthritis with synthetic and biological disease-modifying antirheumatic drugs: 2016 update. Ann Rheum Dis. 2017;76:960-77. 10.1136/annrheumdis-2016-210715 28264816

