# Supplementary material for: The role of anti-citrullinated protein antibody reactivities in an inception cohort of patients with rheumatoid arthritis receiving treat-to-target therapy
Source: Arthritis Res Ther. 2018 Jul 13;20:146. doi: 10.1186/s13075-018-1635-7 (PMC6044041; doi:10.1186/s13075-018-1635-7)
Supplement: Supplementary file 1 — Table S1. Disease characteristics (median values) in ACPA reactivity-positive versus reactivity-negative patients in the anti-CCP-positive sub-group (n = 178); p values were derived from the Mann-Whitney U test and statistically significant differences are marked in bold. Reactivities are sorted by decreasing frequency in the cohort. Table S2. Correlation between ACPA reactivities and disease activity (baseline). Spearman correlation (p value). Table S3. Correlation between reactivities and disease activity in anti-CCP-positive patients (baseline). Spearman correlation (p value). (DOCX 41 kb) [file 13075_2018_1635_MOESM1_ESM.docx]

**SUPPLEMENTARY MATERIAL**

**Table S1:** Disease characteristics (median values) in ACPA reactivity positive vs. negative patients in anti-CCP positive sub-group (n=178), p-values from Mann-Whitney U-test and statistically significant differences marked in bold. Reactivities are sorted by decreasing frequency in cohort.

|  | | **ESR** | | **CRP** | | **DAS** | | **SJC44** | | **RAI** | | **US GS** | | **US PD** | | **vdHSS E** | | **vdHSS JSN** | | **vdHSS T** | |
| --- | --- | --- | --- | --- | --- | --- | --- | --- | --- | --- | --- | --- | --- | --- | --- | --- | --- | --- | --- | --- | --- |
|  | |  | ***p*** |  | ***p*** |  | ***p*** |  | ***p*** |  | ***p*** |  | ***p*** |  | ***p*** |  | ***p*** |  | ***p*** |  | ***p*** |
| **Fibβ 60-74** | **+** | 20 | 0.30 | 7 | 0.13 | 3.26 | 0.66 | 7 | 0.60 | 7 | 0.65 | 17 | 0.63 | 6 | 0.69 | 3 | 0.51 | **1** | **0.01** | 4.5 | 0.08 |
|  | **-** | 24 |  | 13 |  | 3.38 |  | 10 |  | 6 |  | 13 |  | 6 |  | 3 |  | **0** |  | 3 |  |
| **Vim 60-75** | **+** | 21 | 0.91 | 7 | 0.40 | 3.29 | 0.87 | 7 | 0.44 | 6 | 0.70 | 17 | 0.56 | 6 | 1.00 | 3 | 0.93 | 0.75 | 0.49 | 4 | 0.95 |
|  | **-** | 22 |  | 6 |  | 3.44 |  | 11 |  | 7 |  | 14 |  | 6 |  | 3 |  | 1 |  | 4 |  |
| **H4 31-50** | **+** | 21 | 0.74 | 8 | 0.69 | 3.24 | 0.13 | 8 | 0.48 | **6** | **0.03** | 17 | 0.25 | 6 | 0.62 | 3 | 0.57 | 1 | 0.43 | 4.25 | 0.32 |
|  | **-** | 17 |  | 6 |  | 3.66 |  | 8 |  | **11** |  | 14 |  | 7 |  | 2.5 |  | 0.5 |  | 3 |  |
| **CEP-1** | **+** | 21 | 0.39 | 8 | 0.35 | 3.29 | 0.62 | 8 | 0.10 | 6 | 0.79 | 17 | 0.16 | 6 | 0.39 | 3 | 0.59 | 1 | 0.06 | 4.5 | 0.21 |
|  | **-** | 18 |  | 5 |  | 3.25 |  | 7 |  | 7 |  | 13 |  | 5 |  | 3 |  | 0.5 |  | 3 |  |
| **Fil 307-324** | **+** | 21 | 0.52 | 8 | 0.14 | 3.28 | 0.52 | 7 | 0.08 | 7 | 0.96 | 17 | 0.75 | 6 | 0.54 | 3 | 0.77 | 1 | 0.20 | 4 | 0.50 |
|  | **-** | 18 |  | 5 |  | 3.31 |  | 11 |  | 7 |  | 16 |  | 7 |  | 3 |  | 0.5 |  | 4 |  |
| **Fibα 573** | **+** | 22 | 0.30 | 8 | 0.46 | 3.27 | 0.55 | 7 | 0.47 | 6 | 0.35 | 17 | 0.36 | 6 | 0.72 | 3 | 0.59 | **1** | **0.03** | 4.5 | 0.16 |
|  | **-** | 17 |  | 6 |  | 3.31 |  | 8 |  | 7 |  | 14 |  | 7 |  | 3 |  | **0.5** |  | 4 |  |
| **Fibβ 36-52** | **+** | 22 | 0.23 | 8 | 0.29 | 3.26 | 0.47 | 8 | 0.86 | 6 | 0.12 | 17 | 0.41 | 6 | 0.80 | 3 | 0.41 | **1** | **<0.01** | **4.5** | **0.04** |
|  | **-** | 18 |  | 6 |  | 3.41 |  | 7 |  | 9 |  | 14 |  | 6 |  | 3 |  | **0** |  | **3** |  |
| **H3 1-30** | **+** | 18 | 0.36 | 7 | 0.78 | 3.29 | 0.64 | 8 | 0.40 | 6 | 0.60 | 17 | 0.56 | 6 | 0.75 | 3 | 0.77 | 0.5 | 0.16 | 4 | 0.33 |
|  | **-** | 22 |  | 7 |  | 3.28 |  | 7 |  | 7 |  | 15 |  | 6 |  | 3 |  | 1 |  | 4.5 |  |
| **H4 14-34** | **+** | 21 | 0.21 | 8 | 0.37 | 3.27 | 0.73 | 7 | 0.68 | 6 | 0.85 | **17** | **0.05** | 7 | 0.06 | 3 | 0.52 | 1 | 0.74 | 4 | 0.5 |
|  | **-** | 18 |  | 6 |  | 3.31 |  | 8 |  | 7 |  | **14** |  | 5 |  | 3 |  | 1 |  | 4.5 |  |
| **H3 21-44** | **+** | 23 | 0.14 | 8 | 0.08 | 3.31 | 0.47 | 9 | 0.57 | 7 | 0.57 | 17 | 0.18 | 6 | 0.41 | 3 | 0.95 | 0.5 | 0.36 | 4 | 0.56 |
|  | **-** | 18 |  | 5 |  | 3.26 |  | 7 |  | 6 |  | 15 |  | 6 |  | 3 |  | 1 |  | 4.25 |  |
| **Fibα 621-635** | **+** | 22 | 0.85 | 8 | 0.85 | 3.29 | 0.45 | **7** | **0.04** | 7 | 0.84 | 16 | 0.69 | 6 | 0.69 | 2.75 | 0.56 | 0.75 | 0.70 | 4 | 0.58 |
|  | **-** | 20 |  | 7 |  | 3.27 |  | **10** |  | 6 |  | 17 |  | 6 |  | 3 |  | 1 |  | 4.25 |  |
| **Vim 2-17** | **+** | 19 | 0.92 | 7 | 0.87 | 3.29 | 0.90 | 7 | 0.17 | 7 | 0.21 | 17 | 0.54 | 6 | 0.90 | 3 | 0.55 | 1 | 1.00 | 4 | 0.60 |
|  | **-** | 21 |  | 7 |  | 3.29 |  | 9 |  | 6 |  | 15 |  | 6 |  | 3 |  | 1 |  | 4 |  |
| **Fibα 36-50** | **+** | 20 | 0.81 | 8 | 0.50 | 3.29 | 0.50 | 8 | 0.97 | 7 | 0.17 | 17 | 0.75 | 6 | 0.57 | 3 | 0.93 | 0.5 | 0.61 | 4 | 0.84 |
|  | **-** | 21 |  | 6 |  | 3.29 |  | 7 |  | 6 |  | 16 |  | 6 |  | 3 |  | 1 |  | 4 |  |
| **Fibα 591** | **+** | 20 | 0.71 | 6 | 0.81 | 3.29 | 0.45 | 7 | 0.15 | 6 | 0.61 | 17 | 0.24 | 6 | 0.48 | 3 | 0.84 | 1 | 0.16 | 4.5 | 0.58 |
|  | **-** | 21 |  | 8 |  | 3.28 |  | 9 |  | 7 |  | 15 |  | 6 |  | 3 |  | 0.5 |  | 4 |  |
| **Fibβ 74** | **+** | 27 | 0.06 | 9 | 0.35 | 3.25 | 0.80 | 8 | 0.82 | 8 | 0.55 | 16 | 0.45 | 6 | 0.88 | 2.5 | 0.58 | 0.5 | 0.56 | 4 | 0.65 |
|  | **-** | 18 |  | 7 |  | 3.29 |  | 7 |  | 6 |  | 17 |  | 6 |  | 3 |  | 1 |  | 4 |  |
| **Fibβ 72** | **+** | 30 | 0.10 | 6 | 0.95 | 3.52 | 0.24 | 9 | 0.37 | 9 | 0.43 | 22 | 0.14 | **9** | **0.03** | 2.75 | 0.62 | 0.5 | 0.87 | 3.75 | 0.72 |
|  | **-** | 20 |  | 7 |  | 3.27 |  | 7 |  | 6 |  | 15 |  | **6** |  | 3 |  | 1 |  | 4 |  |

Abbreviations: ESR, erythrocyte sedimentation rate (mm/hour, 1-140); CRP, C-reactive protein, mg/L; DAS, Disease Activity Score (0-10); SJC, swollen joint count (0-44); RAI, Ritchie Articular Index (0-78); US, ultrasound; GS, grey scale (0-96); PD, power Doppler 0-96); vdHSS, van der Heijde Sharp score; E, erosion (0-280); JSN, joint space narrowing (0-168); T, total (0-448); *p*, *p* value, Fib, fibrinogen; Vim, vimentin; H, histone; CEP-1, citrullinated enolase peptide-1; Fil, filaggrin; numbers referring to amino acid sequence

**Table S2:** Correlation between ACPA reactivities and disease activity (baseline). Spearman correlation (p-value).

| **Reactivity** | **DAS** | **SJC44** | **ESR** | **CRP** | **US GS** | **US PD** |
| --- | --- | --- | --- | --- | --- | --- |
| Anti-CCP | -0.24 (0) | -0.39 (0) | 0.06 (0.41) | -0.09 (0.2) | -0.25 (0) | -0.2 (0) |
| Fibβ 60-74cit | -0.2 (0) | -0.32 (0) | 0.01 (0.84) | -0.1 (0.13) | -0.24 (0) | -0.21 (0) |
| Vim 60-75cit | -0.1 (0.16) | -0.23 (0) | 0.08 (0.24) | -0.03 (0.61) | -0.24 (0) | -0.17 (0.01) |
| H4 31-50cit | -0.16 (0.02) | -0.31 (0) | 0.07 (0.29) | -0.04 (0.57) | -0.14 (0.04) | -0.14 (0.04) |
| CEP-1 | -0.1 (0.15) | -0.19 (0.01) | 0.1 (0.12) | 0 (0.98) | -0.18 (0.01) | -0.15 (0.03) |
| Fil 307-324cit | -0.19 (0) | -0.37 (0) | 0.07 (0.29) | -0.02 (0.74) | -0.19 (0.01) | -0.16 (0.02) |
| Fibα 573cit | -0.18 (0.01) | -0.31 (0) | 0.07 (0.29) | -0.03 (0.65) | -0.15 (0.03) | -0.13 (0.06) |
| Fibβ 36-52cit | -0.14 (0.03) | -0.26 (0) | 0.14 (0.05) | 0.02 (0.82) | -0.1 (0.15) | -0.1 (0.15) |
| H3 1-30cit | 0 (0.98) | -0.11 (0.1) | 0.04 (0.59) | -0.02 (0.76) | -0.12 (0.09) | -0.11 (0.11) |
| H4 14-34cit | -0.02 (0.74) | -0.19 (0) | 0.14 (0.04) | 0.05 (0.44) | -0.09 (0.2) | 0 (0.96) |
| H3 21-44cit | -0.02 (0.82) | -0.16 (0.02) | 0.11 (0.12) | 0.06 (0.37) | -0.12 (0.08) | -0.12 (0.09) |
| Fibα 621-635cit | -0.16 (0.02) | -0.3 (0) | 0.06 (0.4) | -0.01 (0.92) | -0.14 (0.04) | -0.12 (0.09) |
| Vim 2-17cit | -0.11 (0.09) | -0.29 (0) | 0.04 (0.55) | -0.06 (0.38) | -0.16 (0.02) | -0.13 (0.06) |
| Fibα 36-50cit | -0.03 (0.69) | -0.12 (0.07) | 0.07 (0.33) | 0.06 (0.39) | -0.09 (0.18) | -0.13 (0.06) |
| Fibα 591cit | -0.2 (0) | -0.24 (0) | -0.04 (0.51) | -0.03 (0.64) | -0.08 (0.23) | -0.04 (0.52) |
| Fibβ 74cit | -0.07 (0.3) | -0.16 (0.02) | 0.12 (0.09) | 0.02 (0.82) | -0.16 (0.02) | -0.06 (0.41) |
| Fibβ 72cit | -0.09 (0.19) | -0.11 (0.11) | 0.08 (0.27) | -0.04 (0.58) | -0.07 (0.32) | 0.01 (0.87) |

Abbreviations: DAS, Disease Activity Score; SJC, swollen joint count; ESR, erythrocyte sedimentation rate; CRP, C-reactive protein; US, ultrasound; PD, power Doppler; GS, grey scale; anti-CCP, anti-cyclic citrullinated peptide; Fil, filaggrin; Vim, vimentin; Fib, fibrinogen; H, histone; CEP-1, citrullinated enolase peptide-1; numbers referring to amino acid sequence

**Table S3**: Correlation between reactivities and disease activity in anti-CCP-positive patients (baseline). Spearman correlation (p-value).

| **Reactivity** | **DAS** | **US GS** | **US PD** | **SJC44** |
| --- | --- | --- | --- | --- |
| Anti-CCP | -0.17 (0.02) | 0.01 (0.88) | -0.04 (0.61) | -0.21 (0.01) |
| Fibβ 60-74cit | -0.12 (0.11) | -0.02 (0.78) | -0.06 (0.39) | -0.14 (0.07) |
| Vim 60-75cit | 0.02 (0.84) | -0.03 (0.65) | -0.04 (0.64) | 0 (0.96) |
| H4 31-50cit | -0.06 (0.41) | 0.14 (0.07) | 0.02 (0.78) | -0.11 (0.13) |
| CEP-1 | 0 (0.99) | 0.07 (0.33) | 0.01 (0.87) | 0.05 (0.55) |
| Fil 307-324cit | -0.12 (0.12) | 0.03 (0.71) | -0.01 (0.85) | -0.23 (0) |
| Fibα 573cit | -0.1 (0.19) | 0.06 (0.45) | -0.01 (0.87) | -0.15 (0.04) |
| Fibβ 36-52cit | -0.05 (0.48) | 0.1 (0.17) | 0.01 (0.92) | -0.1 (0.18) |
| H3 1-30cit | 0.13 (0.09) | 0.09 (0.22) | 0.03 (0.65) | 0.11 (0.14) |
| H4 14-34cit | 0.13 (0.07) | 0.2 (0.01) | 0.21 (0) | 0.08 (0.31) |
| H3 21-44cit | 0.1 (0.2) | 0.1 (0.19) | 0.02 (0.78) | 0.05 (0.51) |
| Fibα 621-635cit | -0.1 (0.19) | 0.02 (0.77) | -0.01 (0.93) | -0.2 (0.01) |
| Vim 2-17cit | 0 (0.95) | 0.06 (0.44) | 0 (0.98) | -0.11 (0.13) |
| Fibα 36-50cit | 0.06 (0.4) | 0.07 (0.34) | -0.03 (0.68) | 0.03 (0.66) |
| Fibα 591cit | -0.15 (0.04) | 0.04 (0.56) | 0.03 (0.71) | -0.15 (0.05) |
| Fibβ 74cit | 0.02 (0.79) | -0.05 (0.54) | 0.01 (0.93) | -0.02 (0.8) |
| Fibβ 72cit | -0.05 (0.48) | -0.01 (0.94) | 0.06 (0.46) | -0.03 (0.68) |
| sum_reactivity | -0.02 (0.83) | 0.09 (0.24) | 0.03 (0.74) | -0.08 (0.28) |

Abbreviations: DAS, Disease Activity Score; US, ultrasound; PD, power Doppler; GS, grey scale; SJC, swollen joint count; anti-CCP, anti-cyclic citrullinated peptide; Fil, fillagrin; Vim, vimentin; Fib, fibrinogen; H, histone; CEP-1, citrullinated enolase peptide-1; numbers referring to amino acid sequence
